# Supplementary material for: Effects of chronic cannabidiol in a mouse model of naturally occurring neuroinflammation, neurodegeneration, and spontaneous seizures
Source: Sci Rep. 2022 Jul 4;12:11286. doi: 10.1038/s41598-022-15134-5 (PMC9253004; doi:10.1038/s41598-022-15134-5)
Supplement: Supplementary file 1 — Supplementary Information 1. [file 41598_2022_15134_MOESM1_ESM.docx]

**
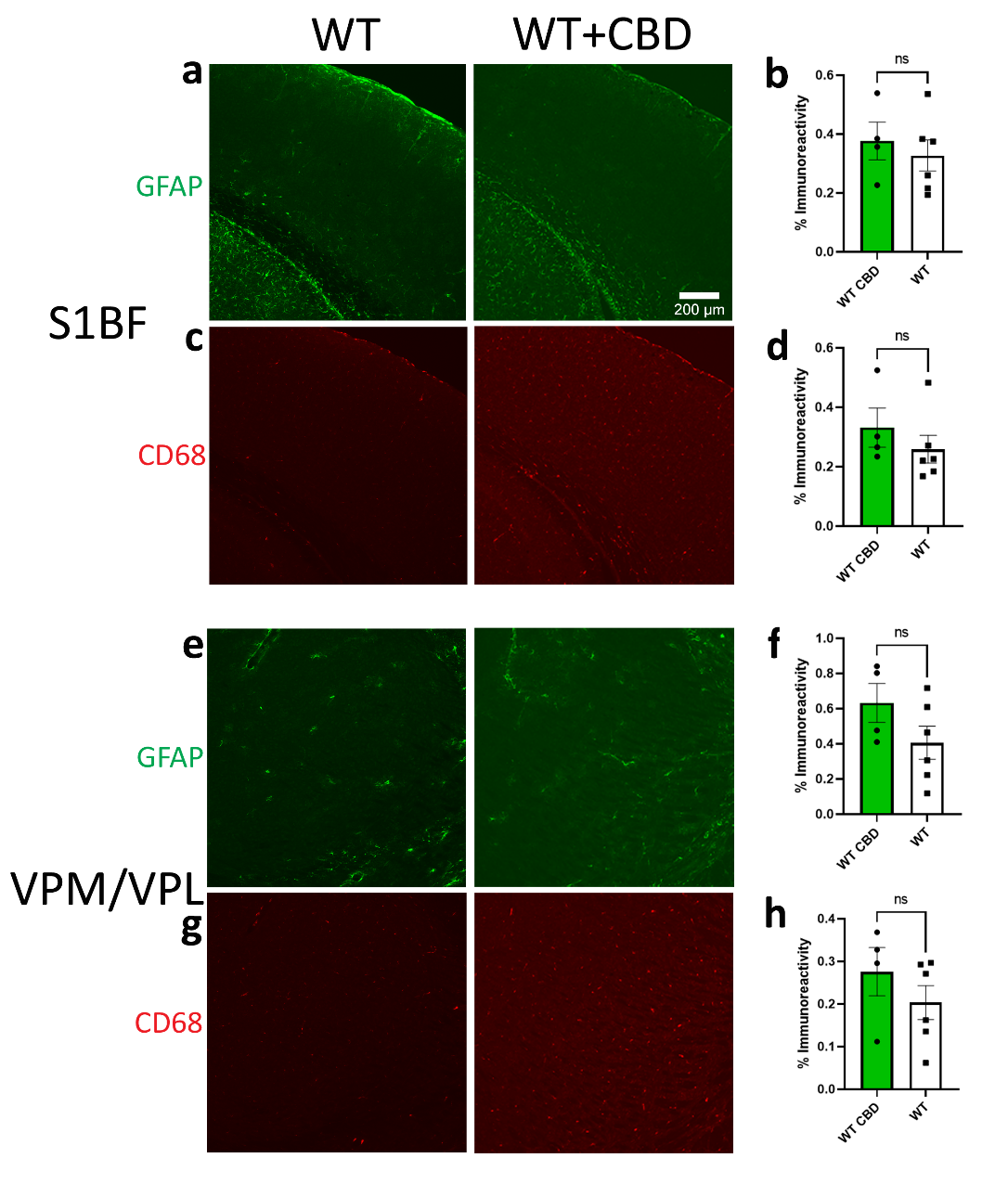
**

**Supplementary Figure S2. Treatment with CBD does not induce pathology in WT mice.** In S1BF, chronic CBD in WT mice did not affect GFAP immunoreactivity (a,b) or CD68 expression (c,d). Additionally, GFAP expression (e,f) and CD68 immunoreactivity (g,h) were unchanged in VPM/VPL. Data shown are means ± SEM. Scale bar is 200 µm.
